# Iridium Oxide Shell Structure on Rutile Titanium Oxide for Efficient Supported Catalyst for the Oxygen Evolution Reaction

**DOI:** 10.1002/advs.202508036

**Published:** 2025-07-26

**Authors:** Elena Cazzulani, Camille Roiron, Lindsay Zhang, Giovanni Ferro, Alasdair Fairhurst, Pierangela Cristiani, Gian Luca Chiarello, Plamen Atanassov

**Affiliations:** ^1^ Department of Chemical and Biomolecular Engineering University of California Irvine CA 92697 USA; ^2^ National Fuel Cell Research Center University of California Irvine CA 92697 USA; ^3^ Horiba Institute for Mobility and Connectivity University of California Irvine CA 92697 USA; ^4^ Department of Chemistry Università degli Studi di Milano Via Camillo Golgi 19 Milano 20133 Italy; ^5^ RSE‐Ricerca sul Sistema Energetico S.p.A. Via Rubattino 54 Milano 20134 Italy

**Keywords:** catalyst, core‐shell structure, iridium loading, oxygen evolution reaction, supported iridium oxide

## Abstract

Proton exchange membrane water electrolysis is a promising technology for the production of hydrogen via water electrolysis. Lower amounts of iridium oxide are needed at the anode to reduce the cost and precious metal dependence of this technology for large‐scale development. The use of iridium oxide catalysts supported on non‐precious supports can help reduce the required iridium loading. This work presents a novel core–shell structure in which a thin layer of IrO₂ is grown on rutile TiO₂ spheres. The structural compatibility between rutile IrO₂ and rutile TiO₂ enables the formation of a continuous IrO₂ shell, unlike on anatase‐rich TiO₂ supports, where only a decorated structure is observed. The core–shell architecture and homogenous IrO₂ distribution on the rutile support is confirmed by imaging, diffraction, and spectroscopy. Electrochemical evaluation demonstrates superior mass activity and pseudo‐capacitance for the core‐shell material (on rutile) than for the decorated structure (on anatase). The core‐shell supported material as electrochemical performance similar to a commercial unsupported IrO₂ catalyst. These results highlight the potential of the rutile IrO_2_‐rutile TiO_2_ interaction for improving catalyst utilization. Such core‐shell materials are promising candidates for an integration in low‐loading systems where unsupported materials suffer in‐plane conductivity losses.

## Graphical abstract

Core–shell and decorated iridium oxide on titanium oxide are formed on spherical supports designed. The core–shell material presents performance similar to that of unsupported materials and does not present the electronic limitations usually present for supported materials with non‐conductive supports.

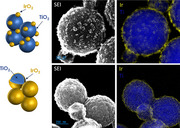

## 1 Introduction

Hydrogen is considered a key energy carrier for the transition to a sustainable energy system. However, most of the current technologies still largely rely on carbon‐based fossil fuels having a significant impact in terms of CO_2_ emissions.^[^ ^1^, ^2^ ^]^ The possibility of a large‐scale adoption of green hydrogen could significantly contribute to the decarbonization of industries and transportation, fostering a cleaner and more efficient energy model.^[^ ^3^, ^4^ ^]^ In this context, the hydrogen economy is gaining interest in the automotive sector, where it can serve as a valid alternative to the highly polluting combustion engine.^[^ ^5^ ^]^ To mitigate environmental impact and support a low‐carbon economy, proton exchange membrane water electrolysis (PEMWE) stands out as the most promising technique for highly pure and efficient hydrogen production from renewable sources.^[^ ^6^, ^7^ ^]^

The main limitation of this process liesin the anodic reaction, which requires a higher overpotential (η) compared to the cathodic reaction (η _anode_ > η _cathode_).^[^ ^8^ ^]^ In fact, the electrochemical splitting of water into oxygen and hydrogen is kinetically hindered by the positive electrode reaction which implies a four‐electron transfer. The oxidized surfaces of the noble metals Ru and Ir are currently considered the most effective electrocatalysts toward the oxygen evolution reaction (OER) due to their high activity and IrO_2_ stability.^[^ ^9^, ^10^, ^11^, ^12^ ^]^

The compromise between activity and durability for the different iridium catalyst surfaces is still the source of research in the community, with amorphous iridium oxide being considered as more active but less durable than rutile iridium oxide.^[^ ^13^, ^14^, ^15^ ^]^ Both structures exhibit sufficiently good properties for implementation in water electrolyzer devices. However, the high cost and scarcity of these precious metals prompt a strong interest in minimizing their content in the membrane electrode assembly.^[^ ^16^ ^]^ The ultimate target loadings for these metals have been set by the American department of energy (DoE) at 0.125 mg_Ir _cm^−2^, while current technologies use loadings in the range of 2 to 3 mg_Ir _cm.^−2^.^[^ ^17^ ^]^

To address this challenge, current strategies focus on designing effective electrodes with catalyst materials presenting favorable morphologies to enhance both utilization and inter‐particle contact, even at low loading. Indeed, when using unsupported iridium oxide catalysts, reducing the catalyst loading leads to in‐plane conductivity issues, lowering the utilization of the iridium. A promising alternative consists in optimizing the geometry of the electrocatalyst by increasing the active layer volume through the addition of a semi‐conductive supporting material. This can lead to the formation of a continuous 3D conductive network. The use of a support allows to increase the ratio of the surface of iridium exposed to the volume of the active layer.^[^ ^18^ ^]^

Titanium dioxide (TiO_2_), also known as titania, is a suitable support for iridium oxide (IrO_2_) in OER applications under acidic conditions.^[^ ^19^, ^20^ ^]^ Titania is a semiconductor that naturally occurs in three crystalline forms: anatase, rutile and brookite, with rutile being the most thermodynamically stable phase.^[^ ^21^ ^]^ The stability and affordability of titanium oxide make it a preferred support for iridium oxide catalysts.^[^ ^22^, ^23^, ^24^, ^25^, ^26^, ^27^, ^28^ ^]^ Iridium nanoparticles can be formed directly on a TiO_2_ support from an iridium salt precursor.^[^ ^22^, ^23^, ^24^ ^]^ In some approaches, the salt is reduced to yield metallic iridium nanoparticles, which are subsequently oxidized in a later stage.^[^ ^22^, ^24^, ^25^, ^26^ ^]^ Alternatively, direct thermal conversion of the salt in air can produce IrO_2_, although this method has predominantly been applied to anatase‐phase titanium oxide,^[^ ^23^, ^27^, ^28^ ^]^ which typically leads to the formation of a discontinuous shell or so‐called decorated structure. In the few cases where rutile TiO_2_ has been employed, the iridium loading is usually very high (50 wt.%), a condition that promotes the formation of iridium oxide nanoparticles rather than the development of a continuous shell structure.^[^ ^28^ ^]^ Notably, both iridium and titanium oxide can crystallize in the rutile structure. Rutile IrO_2_ has lattice parameters (a = b = 4.50 Å, c = 3.16 Å) like those of rutile titania (a = b = 4.59 Å, c = 2.96 Å).^[^ ^29^, ^30^ ^]^ The lattice mismatch is of 2% along the a and b axis and of 6% along the c axis, makes possible the formation of epitaxial heterostructures between these two crystal structures.^[^ ^31^ ^]^ The aim of this work is to explore how the similarities between the crystal structure of the support and the catalyst can promote complete coverage of the semiconductor surface with IrO_2_, leading to the formation of a core‐shell structure.

A sol‐gel synthesis tailored to produce spherical particles of several hundreds of nanometers of diameters and the presence of a surfactant allows the formation of well‐dispersed rutile titania spheres. Calcination under a reducing environment induces the formation of a suboxide layer on the surface of bulk‐rutile titania, enhancing the conductivity of the support.^[^ ^32^ ^]^ The resulting material, known as black TiO_2_ and designated in this paper as TiO_2_‐R, consists of a rutile titania bulk with a suboxide surface. The prepared TiO_2_‐R is used as a substrate for IrO₂ growth, leading to the formation of core‐shell particles (IrO₂@TiO₂‐R). For comparison, the formation of a continuous shell on an anatase‐rich substrate (TiO_2_‐AR) is not achieved.

The formation of the core‐shell structure represents a promising strategy for the efficient utilization of IrO₂ in proton exchange membrane water electrolysis (PEMWE). The electrochemical activity toward the oxygen evolution reaction is evaluated in a liquid electrolyte, using a three‐electrode configuration. The performance compares favorably with that of a commercial unsupported iridium oxide catalyst, with the added advantage that this material could be integrated into a low‐loading membrane electrode assembly (MEA) without expected in‐plane conductivity limitations.

## 2 Results and Discussion

A sol‐gel synthesis of TiO₂ spheres is developed to obtain two supports with the same morphology but different crystal structure. The size and shape of the particles are controlled using tetradecyl amine (TDA) as a surfactant in a solvent mixture of methanol and acetonitrile. A small amount of water is added to the mixture and was preferentially located within the templating micelles formed. Upon addition of titanium (IV) isopropoxide (TTIP), it quickly reacts with the water inside the micelles. The system is aged for 24 h prior to surfactant removal and subsequent drying via freeze‐drying.^[^ ^33^ ^]^ The resulting powder is then subjected to thermal treatment in a reducing atmosphere (5% H_2_/Ar) at different temperatures. The crystalline phase obtained depends on the calcination temperature,^[^ ^34^ ^]^ leading to the formation of anatase and/or rutile titanium oxide.

The two calcination temperatures are designed to obtain rutile pure (800 °C, TiO_2_‐R) and anatase‐rich (550 °C, TiO_2_‐AR) particles. Both obtained structures are black, likely indicating the incomplete oxidation of the titanium lattice. This hypothesis is further supported by thermogravimetric analysis (TGA), as shown in Figure S1 (Supporting Information), which displays the thermogram of TiO_2_‐R. The measurement is conducted in synthetic air over a temperature range of 50–800 °C with a heating rate of 3 °C min^−1^. The analysis reveals a mass gain peaking at 570 °C, corresponding to an increase of 0.7 wt.% relative to the initial mass. This is attributed to the oxidation of the titanium suboxides present on the surface, as reported in previous studies.^[^ ^35^, ^36^, ^37^, ^38^, ^39^ ^]^ The oxidation process is accompanied by a noticeable color change of the powder from black to white after thermal treatment. Assuming a homogeneous distribution of suboxide defects throughout the rutile lattice, the observed mass gain corresponds to 1.6% of oxygen vacancies. Alternatively, considering that the suboxide defects are confined to the surface layer and that the entire mass gain arises from the oxidation of suboxide TiO_(2‐x)_ to TiO_2_, the following Equation (1) can be used to estimate the mass fraction of the material present in suboxide form., the following Equation (1) can help us calculate the mass fraction in suboxide form.

(1) $$\frac{m_{sub}}{m_{ox}}=\frac{1}{\frac{M_{ox}}{M_{sub}\cdot wt{\%}_{gain}}-1}$$

With m_sub_ and m_ox_ the mass of suboxide and oxide respectively, M_sub_ and M_ox_ the molar mass of suboxide and oxide respectively, and wt.%_gain_ the mass gain measured by TGA at 570 °C. Assuming the suboxide is TiO_1.75_ (Ti_4_O_7_), for a mass gain of 0.7%, the m_sub_/m_ox_ ratio is calculated to be 0.6%. This translates into an external suboxide layer of the order of magnitude of 1 nm for a 430 nm oxide particle. It is therefore expected that the suboxide features would not be detectable by X‐ray diffraction (XRD), which primarily probes the bulk of the material.^[^ ^40^, ^41^ ^]^

XRD analysis on TiO_2_‐R (Figure S2, Supporting Information) confirms the presence of a rutile crystalline structure (see ICSD‐80843 reference card). Conversely, the titania sample calcined at 550 °C (TiO_2_‐AR) results in a mixture of anatase (see ICSD‐63711 reference card) and rutile phases. The characteristic peaks at 2θ = 27.4° (110) and 2θ = 25.5° (101) are used to conduct semi‐quantitative analysis to determine the relative ratio between the two structures, which reveals the presence of 74% anatase and 26% rutile.

The mean cohesive length, associated to the crystallite size (D) of both phases, is estimated using the following Scherrer's (Equation 2)^[^ ^42^ ^]^:

(2) $$D=\frac{k\lambda }{\beta \;cos\theta }$$

where β is full width at half maximum (FWHM) measured, subtracted from the instrument broadening (0.18°), and k is Scherrer's constant which is taken as equal to 0.89 for all structures.

The rutile crystallites are measured to be 40 nm in TiO_2_‐R and 18 nm in TiO_2_‐AR, while the anatase crystallites in TiO_2_‐AR are ≈17 nm.

**Figure 1:**
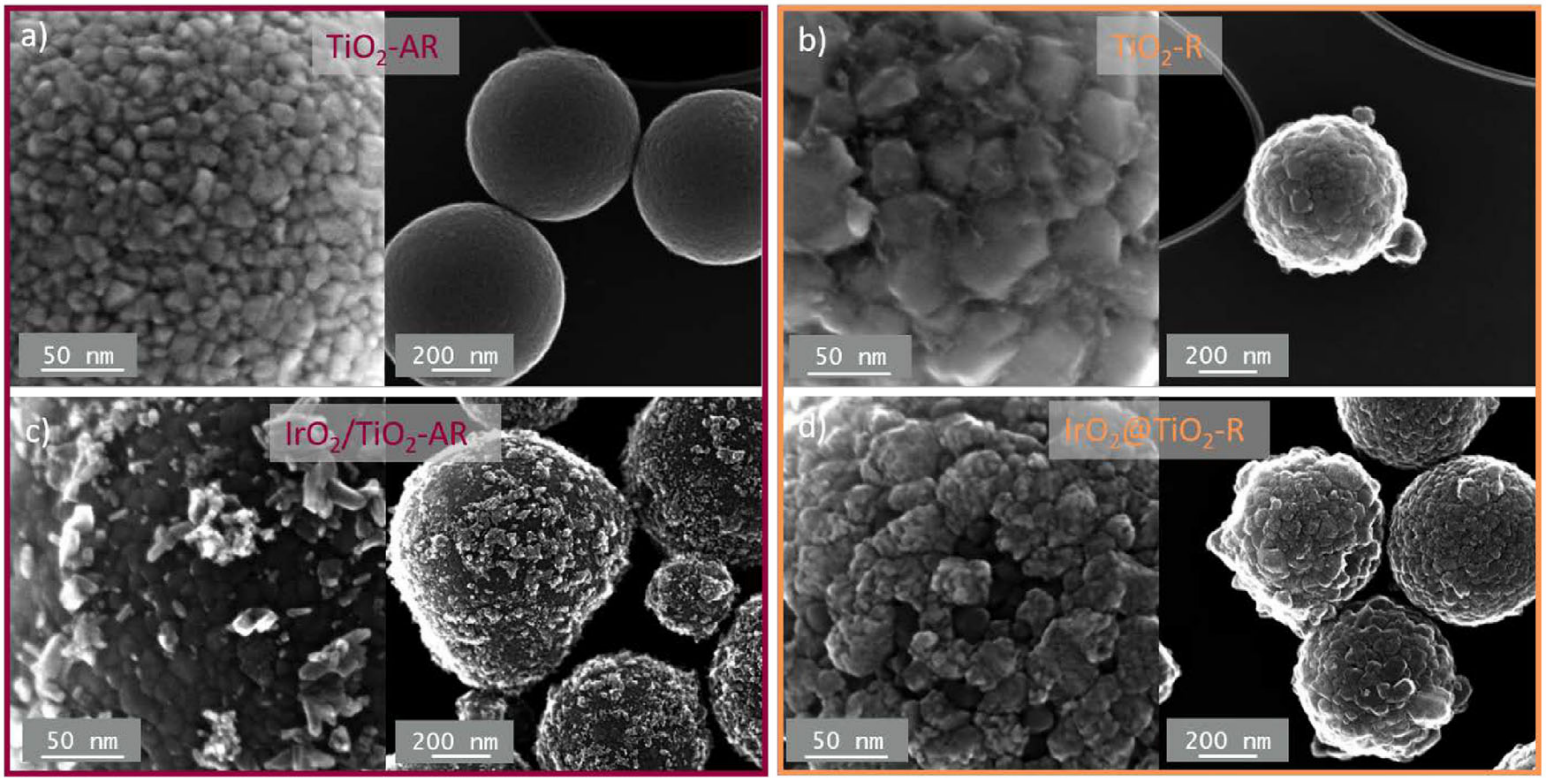
STEM images of a) TiO_2_ spheres calcined at 550 °C, b) TiO_2_ spheres calcined at 800 °C, c) iridium oxide decorated spheres of anatase‐rutile TiO_2,_ and d) core‐shell iridium oxide on TiO_2_ rutile.

The scanning transmission electron microscopy (STEM) images of the bare supports displayed on **Figure** **1**a,b confirm the spherical morphology for both supports. Minimal agglomeration of the spheres is observed, even for TiO_2_‐R calcined at 800 °C. The TiO_2_‐AR particles appear notably smoother than TiO_2_‐R which is consistent with the smaller crystallites measured from XRD patterns. The nanostructures visible on the zoomed‐in views of the support surface is consistent with the titanium oxide crystallite sizes measured by XRD. Particle size distributions for both TiO_2_‐R and TiO_2_‐AR are generated from manual counting on SEM images (Figure S3, Supporting Information). The mean particle diameter for TiO_2_‐R is 432±89 nm and 403±93 nm for TiO_2_‐AR. The two supports provide a good platform to study the effect of the crystal structure on the formation of IrO_2_@TiO_2_ core‐shell particles.

Iridium oxide is deposited onto each of the supports via wet impregnation, starting from a lab‐prepared aqueous solution of IrCl_6_^2−^, followed by thermal treatment in air. The obtained materials are designated as IrO_2_/TiO_2_‐AR and IrO_2_@TiO_2_‐R. For the two catalyst materials, the secondary electron images (SEI) obtained by STEM are displayed on Figure 1b,d. The morphology obtained is significantly different, with the presence of nanoparticles creating roughness at the surface of the anatase‐rich support, while for the pure rutile support, the morphology remains similar to that of the bare support. In the X‐ray diffractogram of the catalyst materials (Figure S2, Supporting Information), the iridium oxide rutile features are negligible compared to the titanium oxide features. The crystal structure of the iridium oxide phase is further investigated in the following discussion. It is worth noting that during the heat‐treatment required to form the iridium oxide, the titanium oxide structures are slightly modified, resulting in an increase in the average crystal size and partial phase transformation of anatase‐TiO_2_ to rutile TiO_2_. For the anatase‐rich support (TiO_2_‐AR), the initial heat‐treatment is performed at 550 °C in reducing atmosphere (5% H_2_/Ar), followed by a second calcination at a slightly lower temperature (500 °C) in air, which could explain the transformation toward the thermodynamically favorable rutile structure. The evolution of the titanium phases is quantified in **Table** **1** for each support.

**Table 1:** Rutile fraction (%) and rutile size (nm) of bare support and iridium.

	TiO_2_‐AR	IrO_2_/TiO_2_‐AR	TiO_2_‐R	IrO_2_@TiO_2_‐R
Rutile fraction [%]	26	43	100	100
Rutile size [nm]	18	21	40	58

The nominal loading is determined based on the average nanoparticle size (Figure S3, Supporting Information), with 8 wt.% of iridium oxide theoretically yielding a shell thickness between 2 and 3 nm, assuming complete coverage of the support. The actual loading is assessed through inductively coupled plasma mass spectrometry (ICP‐MS) after digestion of the IrO_2_/TiO_2_‐AR and IrO_2_@TiO_2_‐R samples. The iridium mass loadings are found to be 4.8±0.5 and 2.6±0.1 wt.% for IrO_2_/TiO_2_‐AR and IrO_2_@TiO_2_‐R, respectively. In the case of IrO_2_@TiO_2_‐R, this would indicate a shell thickness of 0.7 nm, assuming complete coverage. The obtained loadings are lower than the nominal values, likely due to the impregnation procedure used. Notably, the amount of iridium salt left for IrO_2_@TiO_2_‐R is qualitatively higher than for IrO_2_/TiO_2_‐AR, which likely explains the difference in final loading.

**Figure 2:**
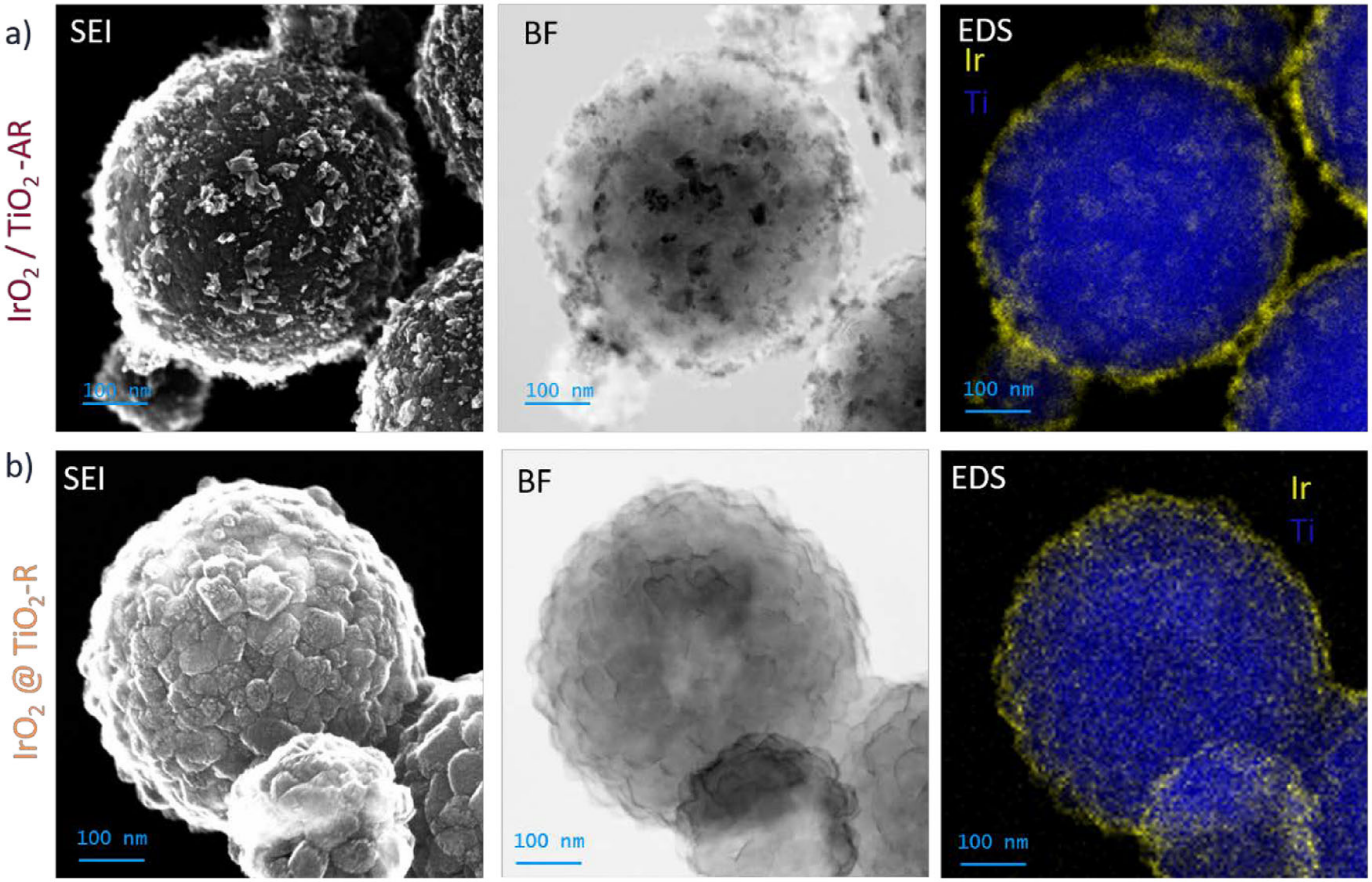
STEM imaging with secondary electron image (SEI) and bright field images (BF) and EDS mapping of Ir K edge and Ti M_5_ edge for a) IrO_2_/TiO_2_‐AR and b) IrO_2_@TiO_2_‐R.

**Figure 3:**
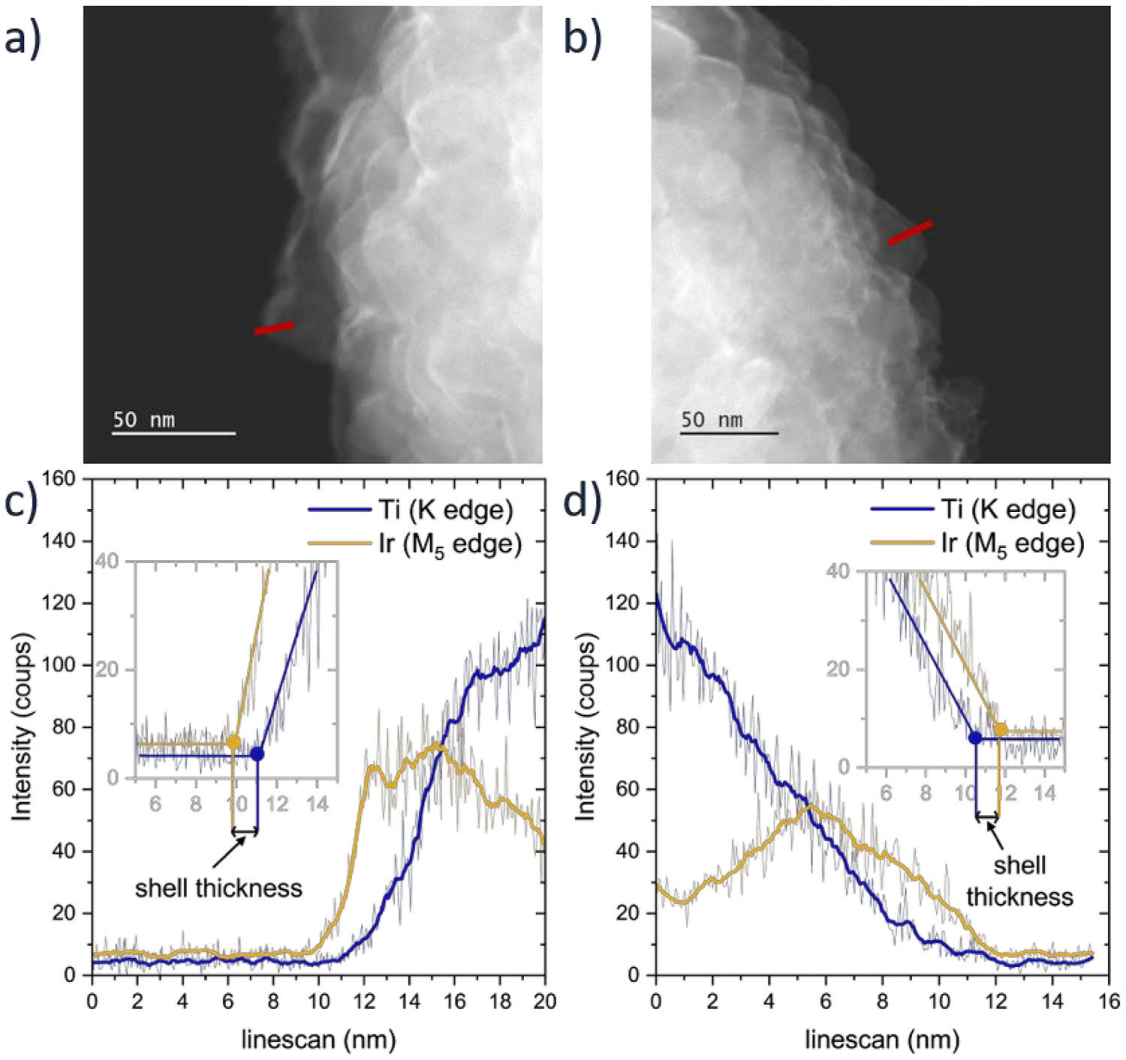
a,b) STEM bright field image and c,d) corresponding line scans from EDS for two zones of IrO_2_@TiO_2_‐R.

The morphology and architecture of the supported catalysts are assessed via scanning transmission electron microscopy (STEM) displayed in **Figure** **2**. The secondary electron images (SEI) provide a 3D view of the material surfaces. The bright field images, resulting from transmitted electrons, offer an atomic weight contrast. For IrO_2_@TiO_2_‐R, the surface of the particle appears with homogeneous contrast, whereas for IrO_2_/TiO_2_‐AR, the nanoparticles forming the surface roughness consist of heavier atoms, likely iridium. This is confirmed by the energy‐dispersive spectroscopy (EDS) mappings of titanium and iridium. The nanoparticles present at the surface of IrO_2_/TiO_2_‐AR are confirmed to be iridium oxide, whereas for IrO_2_@TiO_2_‐R, the iridium signal is strong and homogeneous at the edge, indicating a core‐shell structure. In conjunction with the absence of visible iridium oxide nanoparticles at the surface of the particle, this indicates a complete coverage of the rutile supports, leading to the formation of IrO_2_@TiO_2_‐R core‐shell particles. In contrast, IrO_2_/TiO_2_‐AR exhibits a decorated morphology with particles of several nanometers visible at the surface. The only difference between the two supports is the crystal structure. Such a difference in the morphology of the iridium oxide at the surface indicates some positive interaction between rutile TiO_2_ and rutile IrO_2_, facilitating the formation of an iridium oxide shell on the support. The formation of an epitaxial heterostructure between the two rutile crystals is therefore likely, with rutile titanium oxide acting as a template for the thermal formation of iridium oxide. However, a direct proof of the interaction at the atomic level would require thin film studies and is not the scope of this study. A direct comparison of the titanium oxide supports with and without iridium oxide is displayed in Figure S4 (Supporting Information) with transmission electron microscopy (TEM). The presence of a thin layer of iridium oxide creates a darker edge on TEM images at high magnification that is not present for the bare support. However, for the anatase‐rich support, the iridium oxide can be clearly identified at the surface of the sphere in the form of small nanoparticles. Energy dispersive spectroscopy line scans are performed on the edge of the spheres to quantify the iridium oxide shell thickness. Two examples are presented in **Figure** **3**. The area used for the scan is very small to ensure that only one TiO_2_ rutile crystal is counted at a time. The increase in intensity for iridium starts before that of titanium, indicating that the very edge of the material is composed solely of iridium. The thickness of the layer is estimated by comparing the position where the iridium signal and titanium signals increase. Using this method, the layer thickness is estimated between 1 and 2 nm, which is in the range of the calculated layer of 0.7 nm for the measured loading.

**Figure 4:**
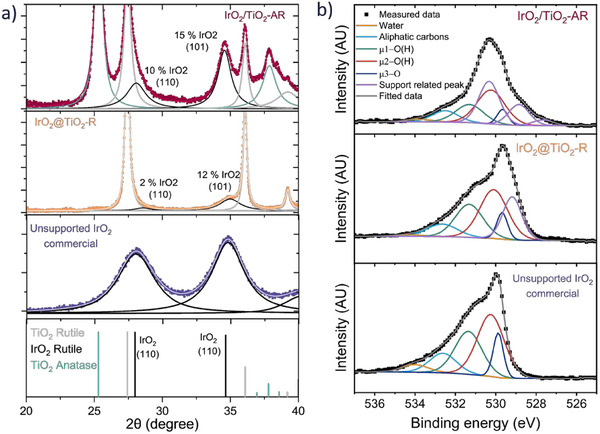
a) X‐Ray diffractograms of IrO_2_/TiO_2_‐AR, IrO_2_@TiO_2_‐R and commercial IrO_2_ unsupported rutile, b) O1s spectra and corresponding fine analysis of IrO_2_/TiO_2_‐AR, IrO_2_@TiO_2_‐R and IrO_2_ unsupported rutile TKK.

The crystallinity of the IrO_2_ is further studied by XRD and X‐Ray Photoelectronic Spectroscopy (XPS). The X‐ray diffractograms of the catalysts are displayed on **Figure** **4**a, with a zoom in the 2θ range between 20 and 40° to highlight the iridium oxide features. A commercial reference of pure iridium oxide rutile material is used as a comparison. The iridium oxide rutile features are deconvoluted using pseudo‐Voigt functions. The (110) diffraction plane is barely distinguishable for IrO_2_@TiO_2_‐R, whereas the (101) plane shows a more distinct feature. Using the semi‐quantitative analysis method with relative intensity ratios (RIR) of 7.9, 5.1, and 4.0 for iridium oxide rutile, titanium oxide anatase, and titanium oxide rutile, respectively. A more in‐depth description of this method is available in our previous work.^[^ ^43^ ^]^ The calculated loading obtained from the (110) and (101) features are very different, especially for IrO_2_@TiO_2_‐R, where the calculated loading varied between 2 and 12%. This suggests an underrepresentation of the (110) plane, pointing toward a preferential crystalline orientation for the iridium oxide shell layer. The (110) facet is expected to be exposed, and this crystal plane is barely present in the diffractogram, whereas the (101) plane is stronger than for an anisotropic crystal. It is difficult to assess whether this preferential exposure of the (110) facet is already present on the titanium oxide rutile support since the crystals are much larger and therefore could not provide this signature in XRD. Applying the Scherrer equation on such convoluted peaks is not suitable and is therefore not attempted.

The iridium oxide structure is further investigated by XPS using a methodology developed by our group to fit the O 1s fine spectra.^[^ ^44^ ^]^ The method is developed on unsupported iridium oxide materials. The iridium layer formed here is thinner than the probing depth of the XPS. As visible in the survey spectra displayed in Figure S5 (Supporting Information), the photoelectrons emitted by the support are detected. Therefore, a contribution of titanium oxide species to the O 1s spectra obtained is expected. The titanium oxide contributions to the O 1s spectra are evaluated by measuring fine spectra for the bare materials (Figure S6a–c, Supporting Information). The contributions are significantly different for the rutile and anatase supports. The main peaks fitted for the bare supports are added to the fitting of the O 1s for IrO_2_/TiO_2_‐AR and IrO_2_@TiO_2_‐R, displayed in Figure 4b. The presence of titanium oxide‐related peaks in the O 1s spectra lowers the reliability of the fittings compared to the unsupported materials. However, the structure obtained is clearly that of a rutile iridium oxide structure for both materials.

**Figure 5:**
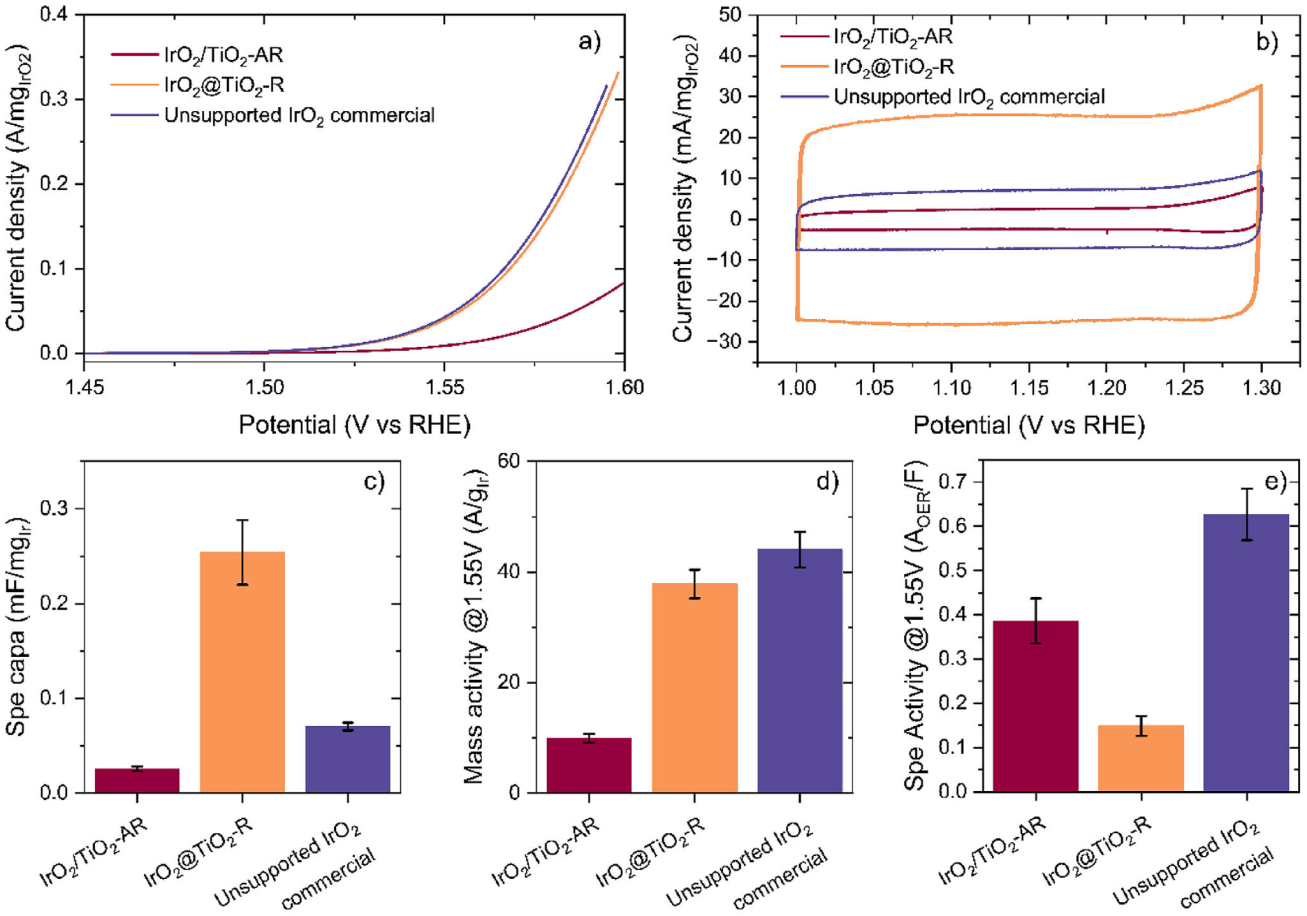
Electrochemical characterization of the iridium oxide catalysts. a) Polarization curves obtained by linear sweep voltammetry at 2 mV s^−1^. The potential is compensated at 85% for iR‐drop, the current is normalized by the loading calculated from ICP‐MS measurements. b) Cyclic voltammetry at 100 mV s^−1^ in a range with minimal redox activity for assessment of the pseudo‐capacitive current. c) Specific capacitance obtained by normalization of the capacitance at 1.1 V by the iridium loading. d) Mass activity obtained from normalization of the current measured at 1.55 V on the LSV by the iridium loading, and e) Specific activity obtained from normalizing the mass activity at 1.55 V by the specific capacitance.

The electrochemical performances of the catalytic materials are evaluated on a rotating disk electrode. A commercial unsupported iridium oxide rutile is used as a benchmark. The electrodes are prepared using a binder‐free ink deposited onto a gold tip. The high frequency resistance is evaluated by electrochemical impedance spectroscopy before each measurement and compensated dynamically at 85% during the measurements, and the 15% left are compensated afterward. The current displayed is normalized by the calculated iridium oxide loading, and all potentials discussed are expressed against the reversible hydrogen electrode (RHE). After activation, cyclic voltammetry (CV) at different sweeping speeds is recorded between 1.0 and 1.3 V to evaluate the pseudo‐capacitive current. The CV at 100 mV s^−1^ are displayed in **Figure** **5**b. First, all materials present limited redox activity with a shape of the CV close to a square in the 1.0 to 1.2 V region. However, the current measured at 1.1 V can only give access to the pseudo‐capacitive current since some redox activity cannot be completely excluded. The core‐shell material IrO_2_@TiO_2_‐R presents a specific pseudo‐capacity significantly higher (0.25 ± 0.03 F/g_IrO2_) than the decorated material IrO_2_/TiO_2_‐AR (0.025 ± 0.002 F/g_IrO2_). Since the iridium oxide was formed in the same conditions in both cases, it is unlikely that this difference would be due to a change of the intrinsic double layer capacitance of the material. This difference is then pointing toward a smaller electrochemically active surface area (ECSA) for IrO_2_/TiO_2_‐AR. This lower ECSA is consistent with the morphology of this material presenting IrO_2_ particles with a low surface to volume ratio and a non‐percolated network of catalyst. On the other hand, for IrO_2_@TiO_2_‐R, the shell structure of the iridium with a thickness smaller than 2 nm allows a good utilization of the iridium. The activity of these materials is assessed by recording a linear sweep voltammetry (LSV) at 2 mV s^−1^ from 1.3 to 1.6 V displayed in Figure 5a. The performance is compared at 1.55 V, in a region where the current is not affected by the elimination of oxygen bubbles during the OER, and expressed as a mass activity in Figure 5d. The decorated material IrO_2_/TiO_2_‐AR presents significantly lower activity than the core‐shell structure IrO2@TiO2‐R, in good agreement with the lower ECSA displayed for this structure. The core‐shell material presents a mass activity similar to that of the unsupported iridium oxide despite showcasing a much smaller capacitive current. This translates for the unsupported material into a much higher specific activity, expressed as the mass activity normalized by the specific pseudo‐capacitance (Figure 5e). This metric is used to characterize the intrinsic catalytic activity of the surface, using the pseudo‐capacitance as a measure of the iridium oxide surface. However, here, the discrepancy is so big between the two specific activities that it would indicate a very different mechanism for the two materials, suggesting very different surface states. This would invalidate the use of the pseudo‐capacitance as a metric of the ECSA that assumes identical specific double‐layer capacitance and surface reactions.

**Figure 6:**
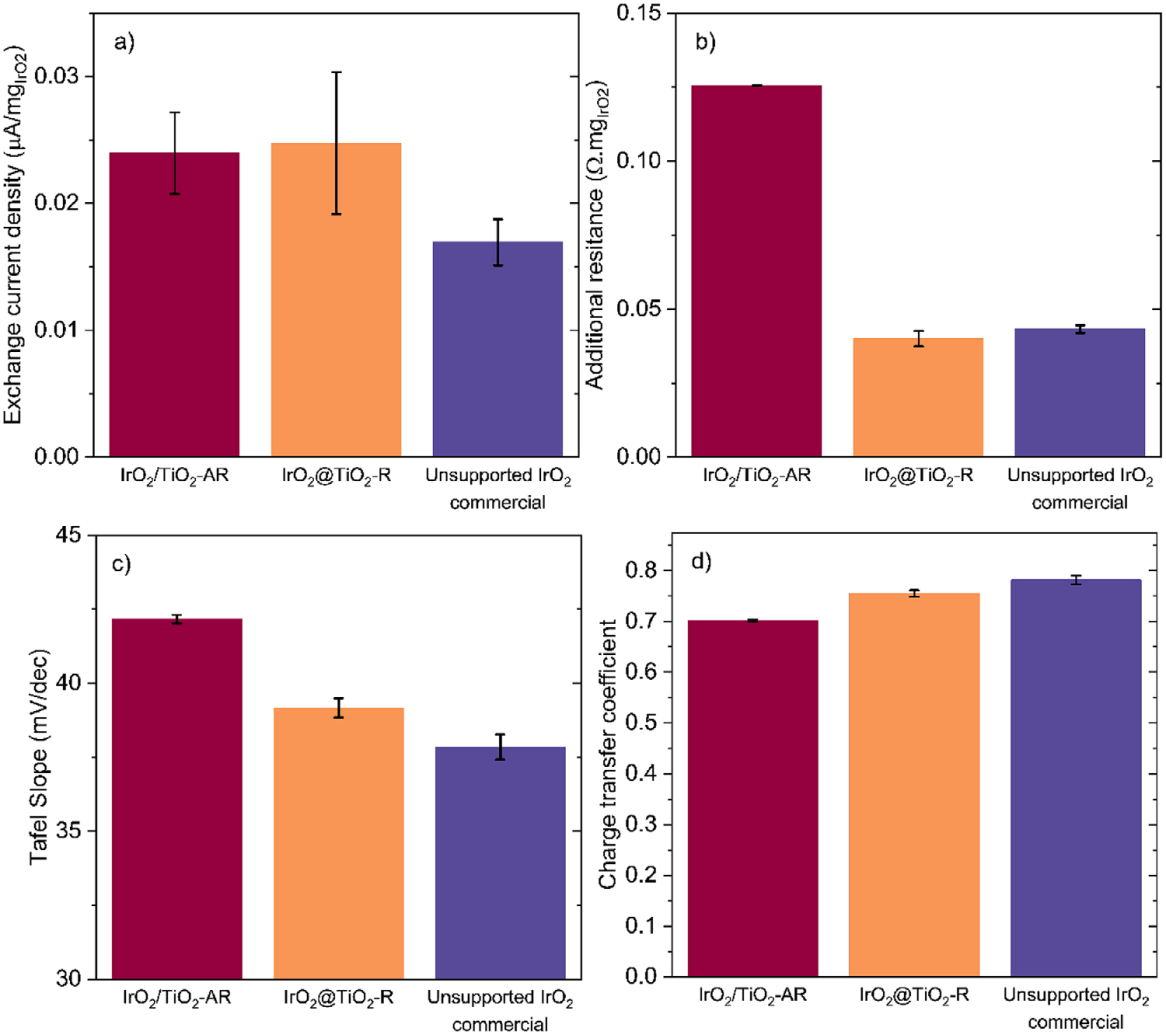
Parameters extracted from the Tafel analysis and non‐linear Butler‐Volmer fitting. a) Mass normalized exchange current density, b) Additional resistance calculated and c) Tafel slope, and d) Charge transfer coefficient.

To further analyze the polarization curves, the methodology described in the Supporting Information was applied to identify discrepancies between the supported and unsupported iridium oxide catalysts. First, the Tafel parameters (Tafel slope and exchange current density) were determined by linear fitting within the optimal overpotential window. These parameters were then incorporated into a Butler–Volmer model to simulate the polarization curve over a broader range. For all materials, the simulated polarization deviated from the experimental data. In all cases, this deviation could be compensated by incorporating an additional resistive contribution into a non‐linear Butler–Volmer fit, while keeping the exchange current density and charge transfer coefficient constant. The resulting Tafel and Butler–Volmer fits for a representative polarization curve of each catalyst are shown in Figure S7 (Supporting Information). The extracted values for exchange current density, Tafel slope, additional resistance, and charge transfer coefficient are presented in **Figure** **6**. Notably, the supported materials in this study exhibited a higher exchange current density than the commercial catalyst (Figure 6a). For the core–shell material, this may indicate improved utilization of the iridium oxide surface, with more active sites per mass of catalyst. However, for the decorated material with larger particles, it is unlikely that catalyst utilization is higher than for the unsupported material. In this case, the higher exchange current density could instead suggest a faster charge transfer at equilibrium. The unsupported material compensates for its slower charge transfer near equilibrium by exhibiting a lower Tafel slope (Figure 6c). This difference is not significant enough to indicate a change in the reaction mechanism (e.g., in the number of electrons in the rate‐determining step) and is therefore only due to changes in the charge transfer coefficient (Figure 6d). As a result, the unsupported material is less efficient near equilibrium but shows increased sensitivity to overpotential for the anodic reaction. The additional resistance calculated for the decorated material is more than three times higher than for the core–shell and unsupported catalysts (Figure 6b). This additional resistance could arise from increased film resistance, mass transport effects, or poor electrical contact between the catalyst and the substrate. Since iridium oxide does not passivate and the catalyst layers are thin (with a 2000 rpm rotation), the most plausible explanation is the latter. Indeed, the iridium oxide support is only weakly conductive. For the decorated catalyst, some iridium oxide nanoparticles are connected only via the titanium oxide support, resulting in higher resistance compared to the core–shell or unsupported materials. The potentiostatic electronic impedance spectroscopy recorded at 1.0 V are modeled to extract the characteristic frequency of the constant phase element for both supported materials (See Supporting Information for methods). The characteristic frequencies obtained are 263 ± 56 and 26 ± 3 Hz for IrO_2_/TiO_2_‐AR and IrO₂@TiO₂‐R, respectively. This difference is highlighted in Figure S8 (Supporting Information) where the imaginary capacitance is plotted against the frequency. The order of magnitude difference confirms the charge accumulation at the surface for the decorated material. The obtention of a continuous shell is therefore a strong advantage for the performance of the material.

Interestingly, the core–shell material exhibits resistive behavior similar to that of the unsupported catalyst, supporting the effectiveness of the core–shell structure. These findings indicate that employing a low iridium oxide loading on a rutile titanium oxide support allows the formation of a core‐shell structure that presents the catalytic properties of unsupported iridium oxide, while significantly reducing the iridium packing density. This characteristic is particularly advantageous for applications in low loading PEMWE.

## 3 Conclusion

This study demonstrates the successful synthesis of core–shell IrO₂@TiO₂‐R structures as highly efficient electrocatalysts for the oxygen evolution reaction (OER). By leveraging the lattice compatibility between rutile TiO₂ and rutile IrO₂, a continuous and uniform IrO₂ shell was formed on the support. In contrast, anatase‐rich TiO₂ supports led to discontinuous decoration with agglomerated IrO₂ nanoparticles. The formation of a thin IrO₂ shell in the core–shell structure enabled high catalyst utilization, resulting in electrochemical performance comparable to that of a commercial unsupported IrO₂ reference. For the decorated material, charge accumulation due to poor conductivity of the support is limiting the activity, but this drawback is not present for the core‐shell material. Overall, this work presents a promising strategy for reducing noble metal usage in PEMWE catalysts by integrating materials engineering and nanoscale design—advancing the development of sustainable hydrogen production technologies.

## 4 Experimental Section

### Materials

Titanium (IV) isopropoxide (TTIP, for synthesis, Technipure) 1‐tetradecylamine (TDA, 95%), methanol (MeOH, ≥99.5%, Emplura), and acetonitrile (ACN, ≥99.9%) were purchased from Sigma Aldrich. Iso‐propyl alcohol (IPA, American Chemical Society grade) was purchased from Macron Fine Chemicals. Perchloric acid solution (70% HClO4, 99.999%, Omnipur) for electrochemical measurements was purchased from Sigma Aldrich. All chemicals were used as received without further purification. Liquid nitrogen was collected from a 22 psi tank from Airgas with 100% purity. Oxygen (ultra high purity, UHP) and 5% H_2_/Ar were purchased from Airgas. Unsupported rutile iridium oxide (TEC77110) was obtained from Tanaka Precious Metals (TKK). Deionized (DI) water was delivered by a Milli‐Q Ultrapure Water System and had a resistivity of 18.2 MW cm at 25 °C.

H_2_IrCl_6_ aqueous solution (IrCl_6_^2−^) was prepared in‐house by digestion of IrCl_3_xH_2_O (99.8%, Sigma Aldrich) in freshly prepared Aqua Regia (3:1 v/v mixture of HCl/HNO_3_ both ACS reagent purity). The solid was digested by heating‐up the dispersion until most of the solid had dissolved. The solution was then filtered using a 100 nm filter syringe to remove all insoluble species. The resulting clear liquid was boiled down until no orange NOx fumes were emitted, even after the addition of excess HCl. The use of a scrubber system was recommended for this step. After the solution had cooled down, an aliquot was quantified by ICP‐MS. The solution used for the synthesis presented here was 45.6 mmol_Ir_/L.

### Synthesis of TiO_2_ Particles

TiO_2_ spheres were prepared via sol−gel synthesis developed from previous works.^[^ ^45^, ^46^, ^47^ ^]^ In a typical procedure, 5 mmol of DI water was added to a 7:3 v/v mixture of MeOH/ACN (25 mL) in a glass vial. Then, 1 mmol of TDA was dissolved in the solution and stirred gently at room temperature for 10 min. The vial was then sealed with a rubber stopper, and 1 mmol of TTIP was added dropwise with a syringe under vigorous stirring. The solution gradually became opaque as TiO_2_ spheres formed. After stirring at room temperature for 24 h, the resulting milky white suspension was centrifugated at 6000 rpm for 20 min, washed with MeOH, and centrifugated again at 6000 rpm for 20 min. It was then washed in water and centrifugated under the same conditions. The so obtained milky gel was redispersed in water, frozen in liquid nitrogen, and subsequently dried under vacuum at room temperature. Finally, the resulting powder was calcined under 5% H_2_ in Ar (300 sccm) at 550 °C (TiO_2_‐AR) and 800 °C (TiO_2_‐R) with a heating ramp rate of 5 °C min^−1^ for 6 h. The resulting materials were cooled naturally in 5% H_2_ in Ar before being exposed to the atmosphere.

### Synthesis of Supported Iridium Oxide Materials

30 mg of TiO_2_ support was added to 4 mL of deionized (DI) water and dispersed in a sonicating bath for 15 min. To further help the dispersion of TiO_2_ spheres, a sonicating horn was used for 5 min following a 2s on and 3s off pulsing cycle at 20% amplitude. To target a nominal Ir loading of 8 wt.%, 263 µL of the previously described IrCl_6_^2−^ solution was added to the dispersion using a micropipette (8.77 µL of solution per mg of TiO_2_ support). The dispersion was then immediately placed into a sonicating bath for another 15 min and then evaporated overnight at 70 °C in an oven. Once the sample was dry, the powder was collected and heat treated under air at 500 °C for 30 min with a ramp rate of 5 °C min^−1^. The resulting material was washed with 300 mL of boiling water, filtered, and dried at 70 °C.

### Physical Characterization

Transmission electron microscopy (TEM), scanning transmission electron microscopy (STEM), and energy dispersive spectroscopy mapping (EDS) were performed on a JEOL 2800 at an accelerating voltage of 300 kV. The core‐shell structure of IrO_2_@TiO_2_ particles was assessed by STEM, and the shell thickness of the precious metal particles was assessed by performing line‐scans of EDS.

X‐ray diffraction measurements were acquired using a Rigaku SmartLab X‐ray diffractometer with a 2.2 kW Cu K𝛼 source (𝜆 = 0.15405 nm) and a Kβ filter. A zero‐background sample holder was used. Rutile TiO_2_, anatase TiO_2_, and rutile IrO_2_ diffraction peaks were identified using ICSD‐80843, ICSD‐63711, and ICSD‐84577, respectively.

Nanoparticles' shape homogeneity was assessed by scanning electron microscopy (SEM) with a FEI Magellan 400 XHR SEM with an accelerating voltage of 10 kV. TiO_2_ nanoparticle size distributions were obtained from SEM images by measuring at least 100 nanoparticles using Image J. Thermogravimetric analysis (TGA) was performed on a TG 209 F1 Libra from Netzsch. Samples were heated to 800 °C (3 °C min^−1^) in an alumina crucible under synthetic air (10 mL min^−1^) to remove any chemisorbed species and fully oxidize the surface of TiO_2_, resulting in a white powder.

X‐ray photoelectron spectra were measured using a Kratos AXIS Supra spectrometer with a monochromatic Al K‐alpha source and an integral charge neutralizer. For each material, a survey spectrum was recorded between 600 and 20 eV of binding energy with a step size of 1 eV. It was verified that the position of C1s main peak was at a binding energy of 485±1 eV. High‐resolution spectra were recorded with a 0.1 eV step size with 200 ms of acquisition per step, and spectra were averaged over 10 measurements. Iridium 4f was probed by a scan of binding energy between 58 and 72 eV, and oxygen 1s by a scan between 527 and 537 eV. The spectra were fitted using CasaXPS software.

IrO_2_ nominal loading was measured using Inductively Coupled Plasma – Mass Spectroscopy after digestion of the samples. 76 µL of catalyst ink (see preparation below) was deposited and dried in a 100 µL alumina crucible and heated dry on a hot plate resulting in the deposition of 0.373 mg of catalyst per crucible. 30 µL of a saturated aqueous solution of KOH/KNO_3_ was added and dried down. The crucibles were heat treated under air atmosphere with the following ramping protocol: 1) 10 °C min^−1^ to 300 °C, 10 min dwell, 2) 3 °C min^−1^ to 360 °C, dwell 4 h, 3) 5 °C min^−1^ to 425 °C, dwell 15 min. This protocol melted the KOH/KNO_3_ and formed iridium and titanium species soluble in Aqua Regia. The crucibles were put in a glass vial with 3 mL of Aqua Regia and heated up (hot plate at 190 °C) until a clear solution was obtained. The liquid was collected using a pipette, and the vial was rinsed with collection of the rinsing solution. The total mass of liquid obtained was measured and used to calculate the volume of solution to be evaluated by using a PerkinElmer Nexion 5000 ICP‐MS

### Electrochemical Characterization

Electrochemical tests were performed in 0.1 m perchloric acid in a three‐electrode glass cell and a rotating disk electrode from Pine Research. All electrochemical measurements were recorded on a VSP‐300 Potentiostat from Bio‐Logic. The counter electrode was a glassy carbon plate, and the reference electrode a Hydroflex hydrogen electrode (RHE) from Gaskatel. Electrocatalysts were deposited on a 0.196 cm^2^ gold tip with a loading of 0.02 mg_IrO2 _cm^−2^ assuming the nominal loading for all catalysts. The mass activity was later calculated based on the actual loading measured by ICP. Catalysts inks were prepared in isopropyl alcohol to achieve a concentration of 204g_catalyst_ µL^−1^. For each electrochemical experiment, 10 µL of the dispersed ink was dropped onto the polished electrode tip. No ionomer binder was used, and no detachment of the catalyst from the gold tip was observed. The electrolyte was systematically changed between each measured electrode, and the reference electrode was never kept in the electrolyte longer than needed for the experiment. Indeed, pollution from the sample accessing the reference electrode shifted drastically the potential of the RHE and led to considerable overestimation of the catalyst performance. The reference electrode was calibrated against a fresh identical RHE in the same electrolyte before and after each experiment. All potentials were expressed against the reversible hydrogen electrode potential.

For each tip, the measurement started with a potential‐governed electrochemical impedance spectroscopy (PEIS) at 1 V between 100 MHz and 10 Hz. In the Nyquist plot, the interception with the X‐axis was taken as the high‐frequency resistance for the electrode, and an 85% compensation was applied to all the subsequent measurements. The electrodes were activated with 50 cycles of voltammetry (CV) between 0.8 and 1.6 V at 100 mV s^−1^ under 2000 rpm rotation of the disk. 3 cycles of CVs between 1.0 and 1.3 V were recorded at 100, 50, and 20 mV s^−1^ to evaluate the capacitive behavior of the electrode. Lastly, a linear sweep voltammetry (LSV) between 1.3 V and 1.6 V was recorded with a sweeping rate of 2 mV s^−1^ under 2000 rpm rotation to evacuate the O_2_ formed at the surface.

## Conflict of Interest

The authors declare no conflict of interest.

## Supporting information



Supporting Information

## Data Availability

The data that support the findings of this study are available from the corresponding author upon reasonable request.
