# Supplementary material for: Iridium Oxide Shell Structure on Rutile Titanium Oxide for Efficient Supported Catalyst for the Oxygen Evolution Reaction
Source: Adv Sci (Weinh). 2025 Jul 26;12(39):e08036. doi: 10.1002/advs.202508036 (PMC12533317; doi:10.1002/advs.202508036)
Supplement: Supplementary file 1 — Supporting Information [file ADVS-12-e08036-s001.docx]

Supporting Information : Iridium Oxide Shell Structure on Rutile Titanium Oxide for Efficient Supported Catalyst for the Oxygen Evolution Reaction

*Elena Cazzulani^#,^, Camille Roiron^#,^, Lindsay Zhang, Giovanni Ferro, Alasdair Fairhurst, Pierangela Cristiani, Gian Luca Chiarello*, Plamen Atanassov**

# Equal contribution

Camille Roiron, Lindsay Zhang, Giovanni Ferro, Alasdair R. Fairhurst, Plamen Atanassov

Department of Chemical and Biomolecular Engineering, University of California Irvine, California 92697, USA 
E-mail: [plamen.atanassov@uci.edu](mailto:plamen.atanassov@uci.edu)

Camille Roiron, Lindsay Zhang, Giovanni Ferro, Plamen Atanassov

National Fuel Cell Research Center, University of California Irvine, California 92697, USA

Alasdair R. Fairhurst

Horiba Institute for Mobility and Connectivity, University of California Irvine, California 92697, USA

Elena Cazzulani, Gian Luca Chiarello

Department of Chemistry, Università degli Studi di Milano, Via Camillo Golgi 19, 20133 Milano, Italy

Elena Cazzulani, Pierangela Cristiani

RSE-Ricerca sul Sistema Energetico S.p.A., Via Rubattino 54, 20134 Milano, Italy


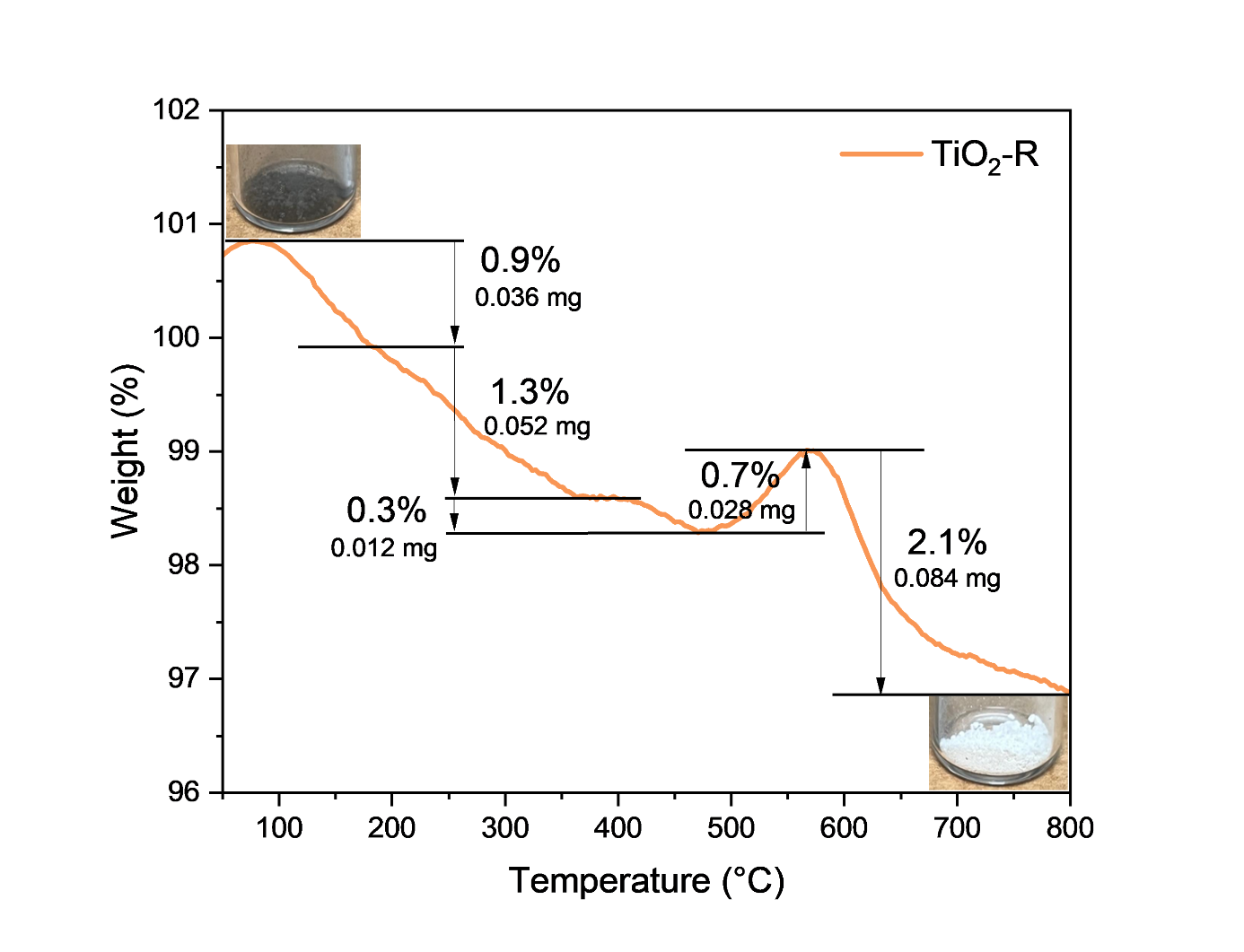


Figure S1. Thermogravimetric analysis (TGA) of TiO_2_-R in synthetic air over the temperature range of 50 - 800°C with a heating rate of 3 °C min^-1^.


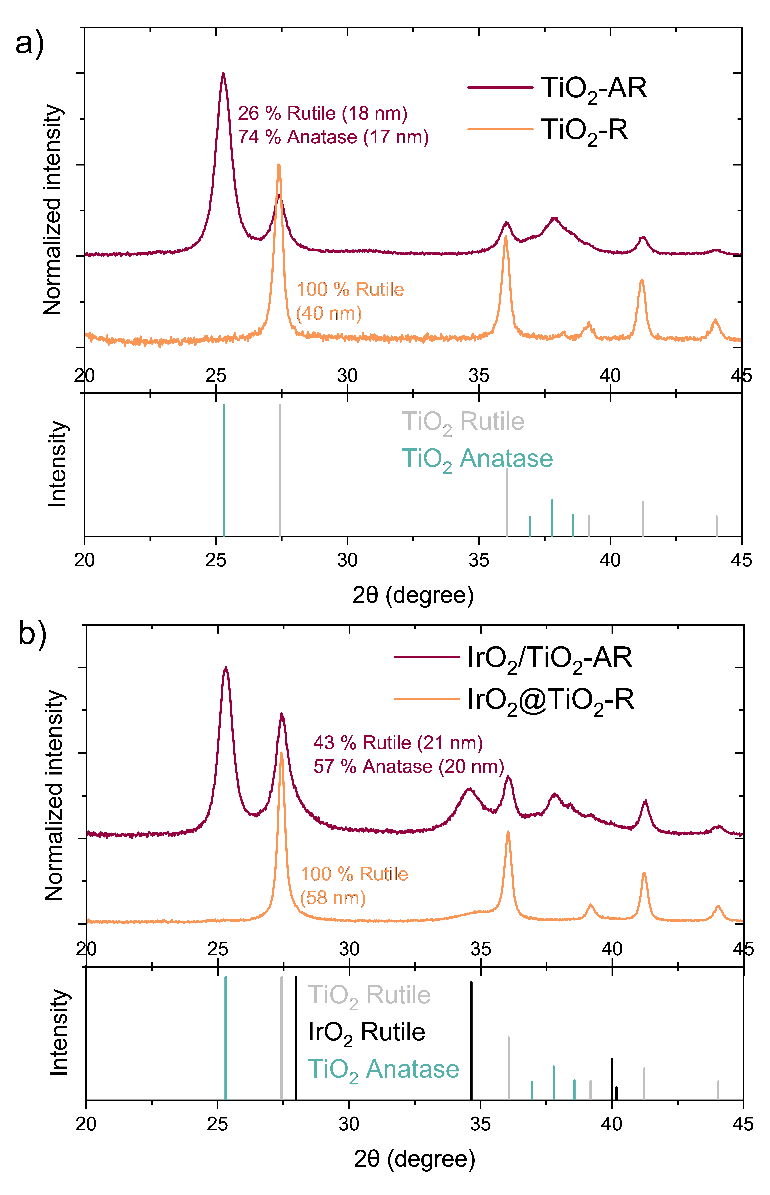


**Figure S2**. XRD patterns of a) the bare titania support materials and b) the final electrocatalysts. Catalysts with titania support in the rutile phase (designated as R) and in both anatase and rutile phase (designated as AR) are shown in orange and red respectively.


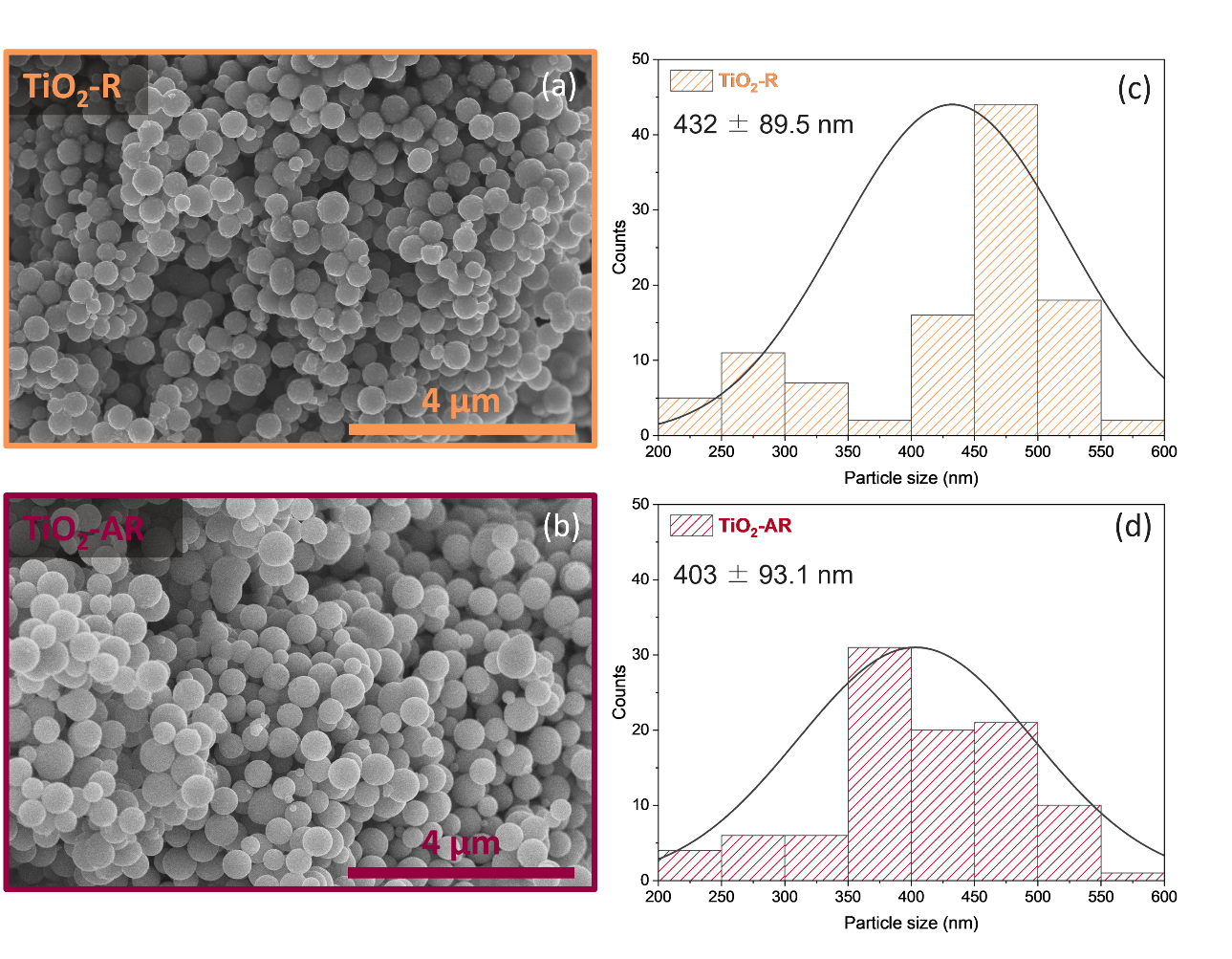


Figure S3. a-b) SEM images of TiO_2_-R and TiO_2_-AR. c-d) Nanoparticles size distribution in evaluated through SEM images. At least 100 particles are measured for each support using ImageJ.


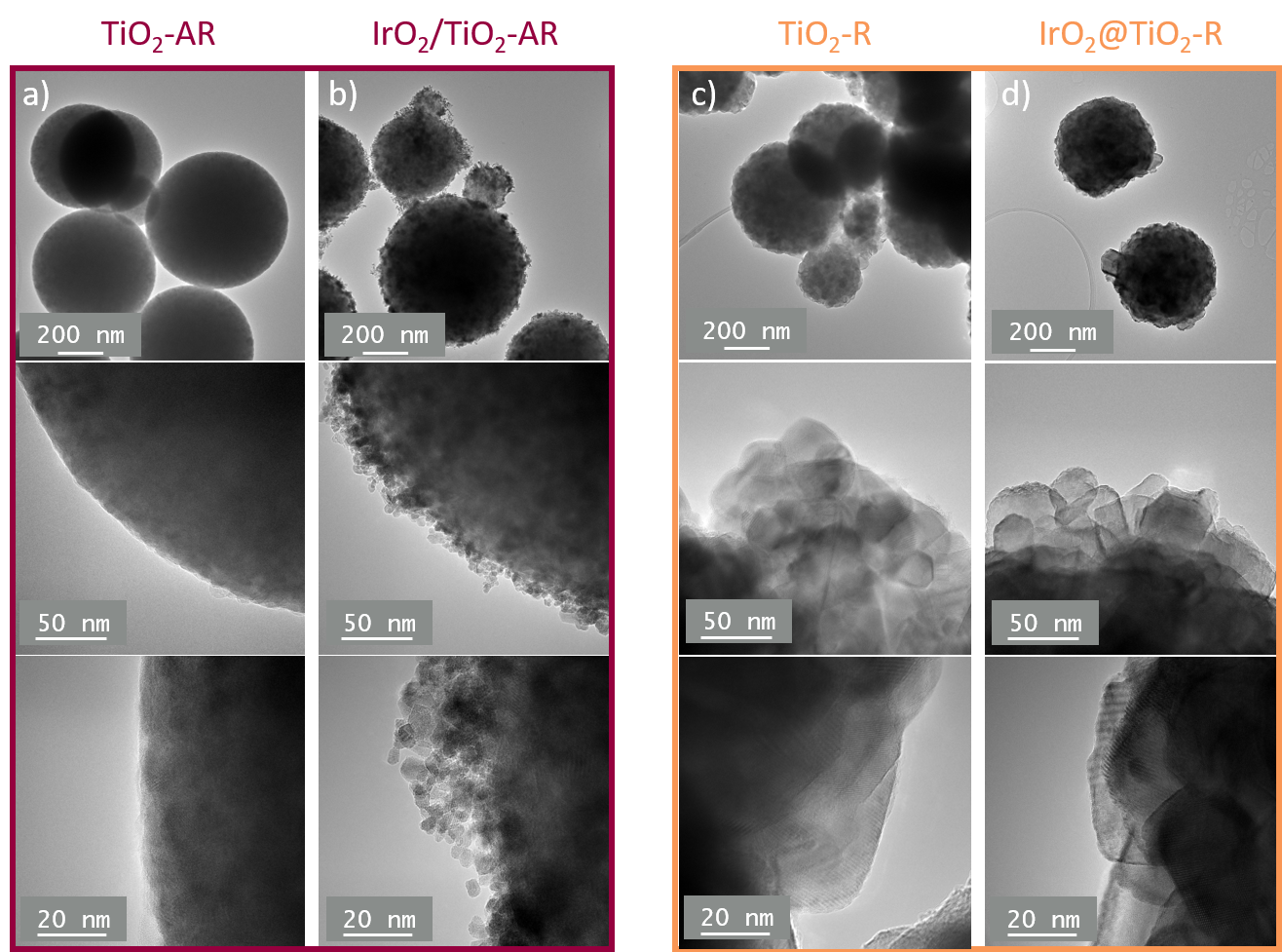


Figure S4. TEM images of a) TiO_2_-AR, b) IrO_2_/TiO_2_-AR, c) TiO_2_-R, and d) IrO_2_@TiO_2_-R.


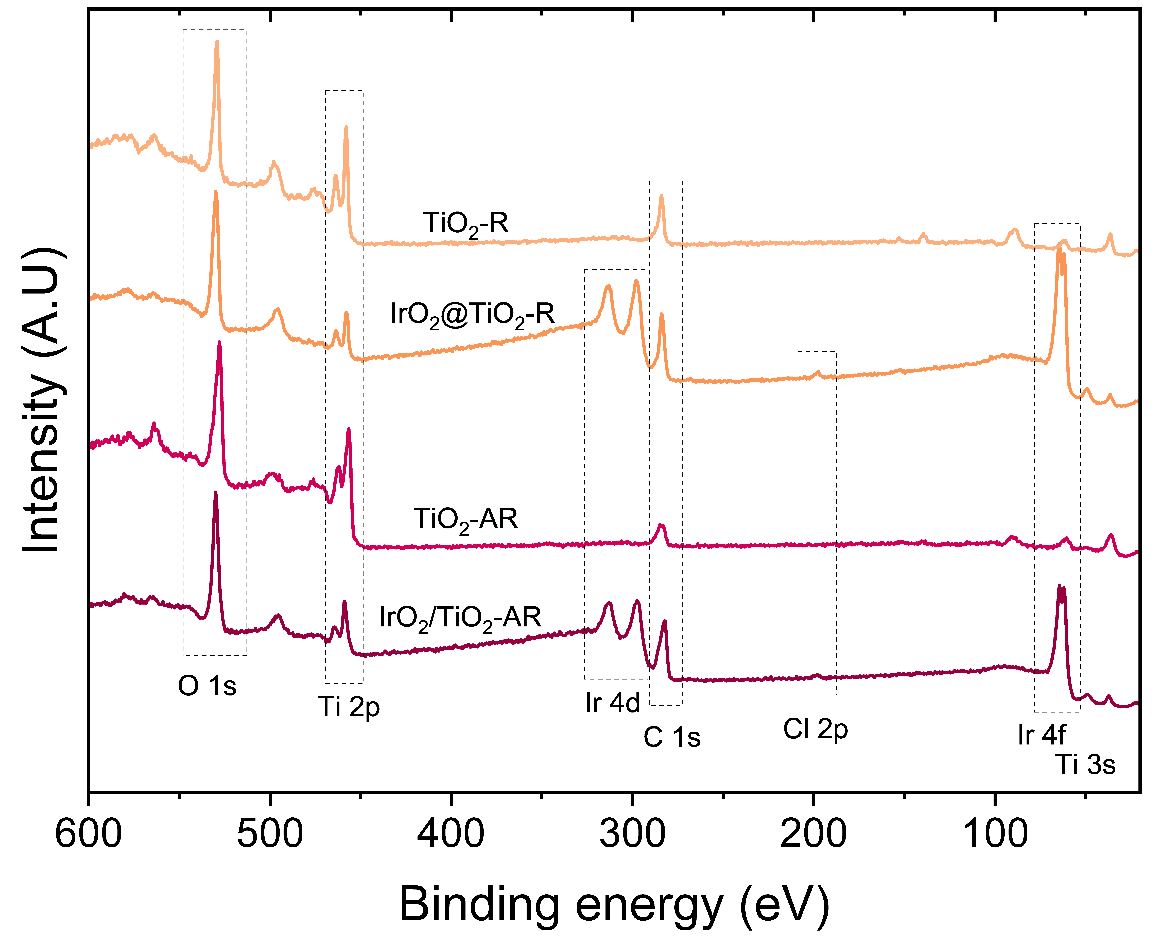


Figure S5. Survey XPS spectra for the bare supports and the IrO_2_/TiO_2_ materials.


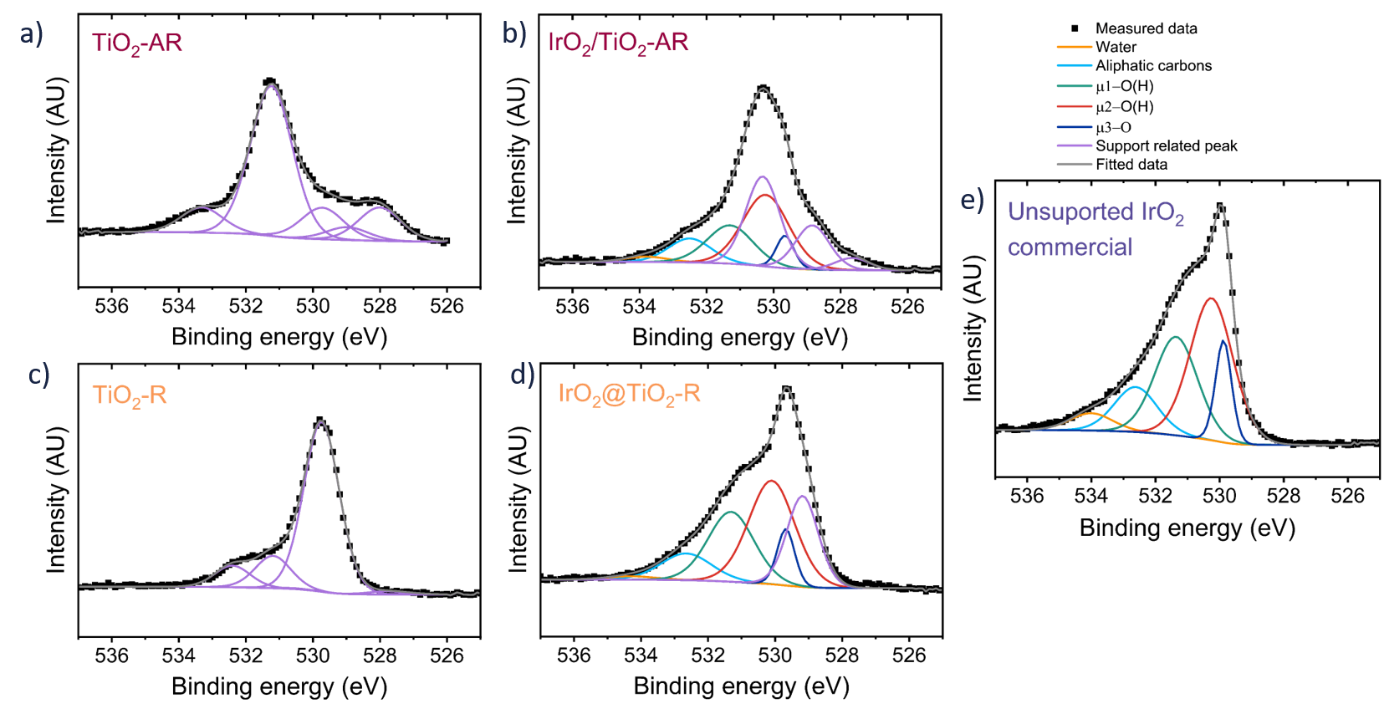


**Figure S6.** O1s spectra and corresponding fine analysis of a) TiO_2_-AR, b) IrO_2_/TiO_2_-AR, c) TiO_2_-R, d) IrO_2_@TiO_2_-R, and e) IrO_2_ unsupported rutile TKK.

Non-linear model for incorporation of an additional resistance.

First, the ohmic resistance is fully compensated (85% in dynamic mode and 15% afterwards), the current between 1.35 and 1.45 V is averaged and subtracted to obtain capacitive current free data. The current is then normalized by the iridium oxide mass loading. The Tafel linear fit is obtained by determination of the best linear potential zone and linear fitting of the current. The current exchange density (mass normalized) and the charge transfer coefficient (assuming a 2-electron limiting step) are extracted (**Equation 1**). They are then incorporated in a Butler Volmer model on the full range of potential screened (**Equation 2**). This step highlights the deviation from the Tafel assumptions at higher potentials. This deviation can be accounted for by incorporating an additional resistance inducing additional overpotential. The modified Butler-Volmer (**Equation 3**) needs to be fitted in a non-linear way. This fitting allows to extract the value of the resistance given in Ω.mg.

*Tafel equation on the anodic branch (OER)*

$\eta\boldsymbol{=}\frac{\boldsymbol{R.T}}{\boldsymbol{\alpha\cdot n\cdot F}}2.303 log(\frac{j}{j_{0}})$ **Equation 1**

- j: IrO_2_ mass normalized current density (A/mg)
- j₀: IrO_2_ mass normalized exchange current density (A/mg)
- η: overpotential (V), defined as E – 1.23 V
- α: charge transfer coefficient (dimensionless)
- n: number of electrons transferred (2 electrons)
- F: Faraday constant (96485 C/mol)
- R: gas constant (8.314 J/mol·K)",
- T: temperature in Kelvin (298 K)

*Linear Butler-vomer equation*

$\boldsymbol{j = j₀ .[}\boldsymbol{exp}^{\frac{\boldsymbol{\alpha\cdot n\cdot F\cdot\eta}}{\boldsymbol{R\cdot T}}}\boldsymbol{-}{\boldsymbol{exp}^{\frac{\boldsymbol{-}\left( \boldsymbol{1-\alpha} \right)\boldsymbol{\cdot n\cdot F\cdot\eta}}{\boldsymbol{R\cdot T}}}}\boldsymbol{]}$ **Equation 2**

- j: IrO_2_ mass normalized current density (A/mg)
- j₀: IrO_2_ mass normalized exchange current density (A/mg)
- η: overpotential (V), defined as E – 1.23 V
- α: charge transfer coefficient (dimensionless)
- n: number of electrons transferred (2 electrons)
- F: Faraday constant (96485 C/mol)
- R: gas constant (8.314 J/mol·K)",
- T: temperature in Kelvin (298 K)

*Non-linear Butler-vomer equation*

$\boldsymbol{j}\boldsymbol{= j₀ .[}\boldsymbol{exp}^{\frac{\boldsymbol{\alpha\cdot n\cdot F\cdot}\left( \boldsymbol{\eta-}\boldsymbol{j}\boldsymbol{\cdot}\boldsymbol{R}_{\boldsymbol{fit}} \right)}{\boldsymbol{R\cdot T}}}\boldsymbol{-}{\boldsymbol{exp}^{\frac{\boldsymbol{-}\left( \boldsymbol{1-\alpha} \right)\boldsymbol{\cdot n\cdot F\cdot}\left( \boldsymbol{\eta-}\boldsymbol{j}\boldsymbol{\cdot}\boldsymbol{R}_{\boldsymbol{fit}} \right)}{\boldsymbol{R\cdot T}}}}\boldsymbol{]}$ Equation 3

- j: IrO_2_ mass normalized current density (A/mg)
- j₀: IrO_2_ mass normalized exchange current density (A/mg)
- η: overpotential (V), defined as E – 1.23 V
- α: charge transfer coefficient (dimensionless)
- R_fit_: Fitted additional resistance (Ω.mg)
- n: number of electrons transferred (2 electrons)
- F: Faraday constant (96485 C/mol)
- R: gas constant (8.314 J/mol·K)",
- T: temperature in Kelvin (298 K)

The potentiostatic electronic impedence spectroscopy at 1.0 V is used to obtain the characteristic frequency of the capacitive behavior of the catalysts. The following model is used with R1 the ionic resistance, CPE1/R2 the charge transfer component and CPE2 the capacitive behavior. The characteristic frequency is calculated using the CPE2 parameters.


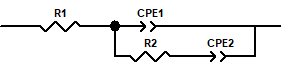


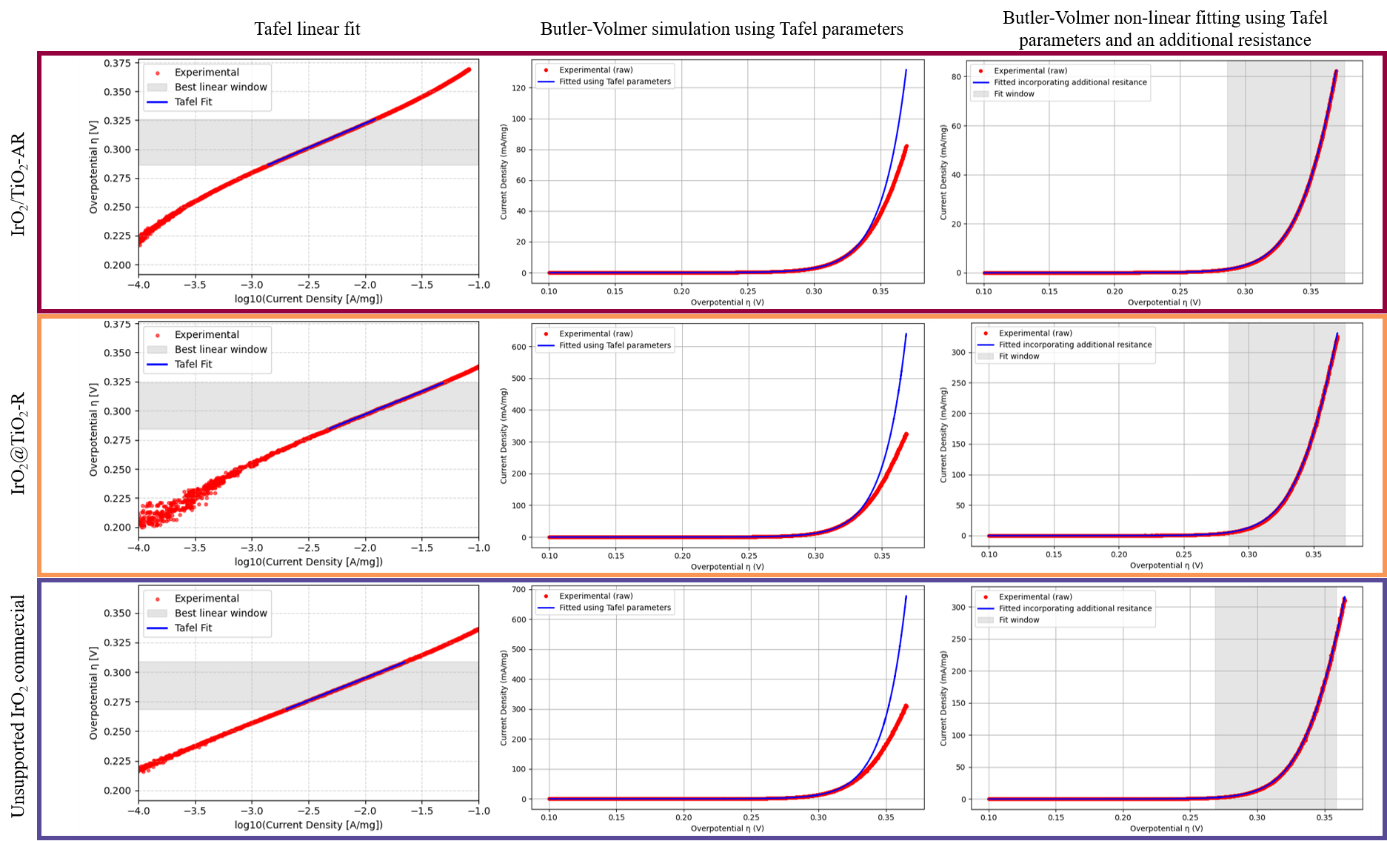


Figure S7. Fitted data using the methodology described above on representative electrodes of each catalysts.


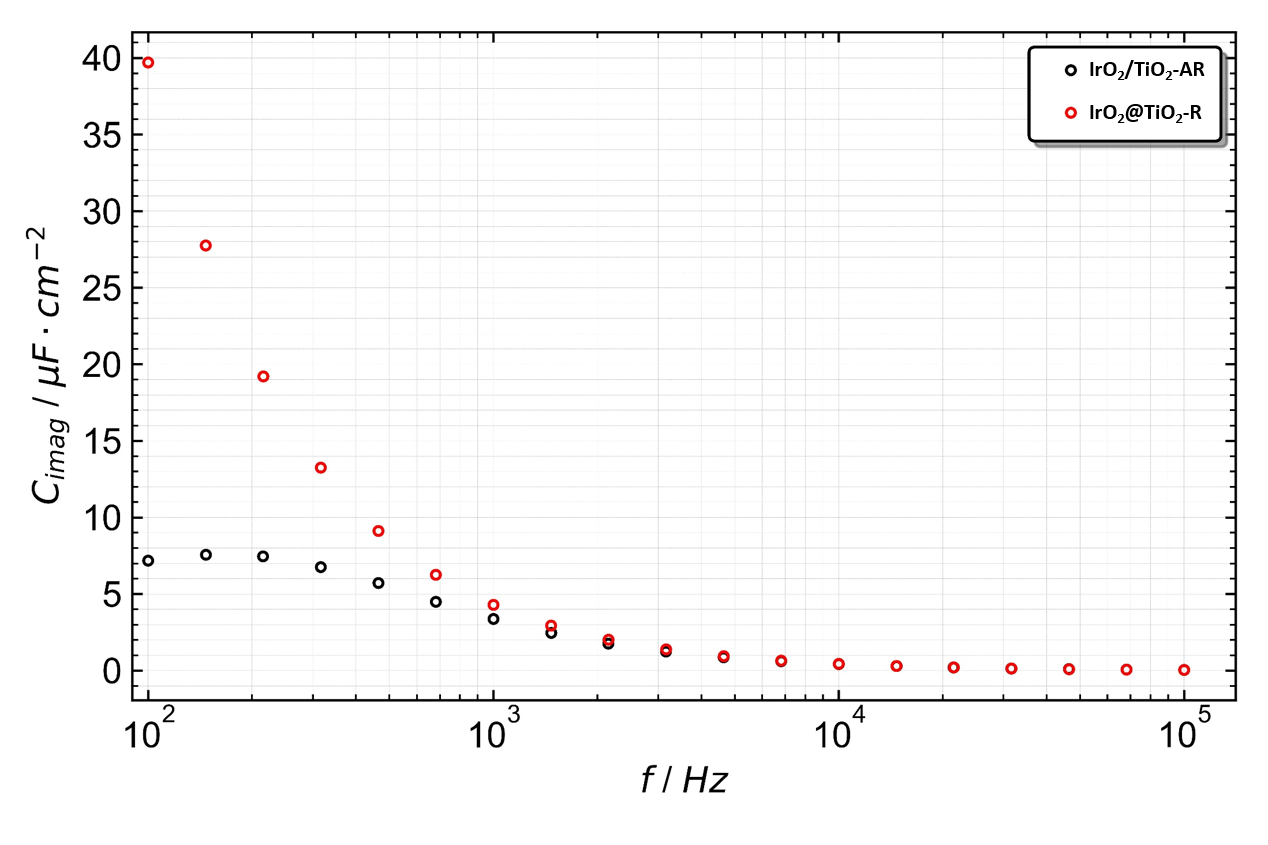


Figure S8. Bode-phase plot of the electronic impedance spectroscopy for the two supported materials. The characteristic frequency could be read as the x-position of the C_imag_ maxima. In the case of the core-shell material it is higher than the scanning range.
